# Effects of α-Adrenoceptor Antagonists on ABCG2/BCRP-Mediated Resistance and Transport

**DOI:** 10.1371/journal.pone.0030697

**Published:** 2012-02-15

**Authors:** Kohji Takara, Kazuhiro Yamamoto, Mika Matsubara, Tetsuya Minegaki, Minoru Takahashi, Teruyoshi Yokoyama, Katsuhiko Okumura

**Affiliations:** 1 Department of Clinical Pharmaceutics, Faculty of Pharmaceutical Sciences, Himeji Dokkyo University, Himeji, Japan; 2 Department of Clinical Pharmacy, Faculty of Pharmaceutical Sciences, Kyoto Pharmaceutical University, Kyoto, Japan; University of Rome, Italy

## Abstract

Acquired resistance of cancer cells to various chemotherapeutic agents is known as multidrug resistance, and remains a critical factor in the success of cancer treatment. It is necessary to develop the inhibitors for multidrug resistance. The aim of this study was to examine the effects of eight α-adrenoceptor antagonists on ABCG2/BCRP-mediated resistance and transport. Previously established HeLa/SN100 cells, which overexpress ABCG2/BCRP but not ABCB1/MDR1, were used. The effects of the antagonists on sensitivity to mitoxantrone and the transport activity of Hoehst33342, both substrates for ABCG2/BCRP, were evaluated using the WST-1 assay and cellular kinetics, respectively. ABCG2/BCRP mRNA expression and the cell cycle were also examined by real-time RT-PCR and flow cytometry, respectively. Sensitivity to mitoxantrone was reversed by the α-adrenoceptor antagonists in a concentration-dependent manner, although such effects were also found in the parental HeLa cells. Levels of ABCG2/BCRP mRNA expression were not influenced by the antagonists. The transport activity of Hoechst33342 was decreased by doxazosin and prazosin, but unaffected by the other antagonists. In addition, doxazosin and prazosin increased the proportion of S phase cells in the cultures treated with mitoxantrone, whereas the other α-adrenoceptor antagonists increased the percentage of cells in G_2_/M phase. These findings suggested that doxazosin and prazosin reversed resistance mainly by inhibiting ABCG2/BCRP-mediated transport, but the others affected sensitivity to mitoxantrone *via* a different mechanism.

## Introduction

Acquired resistance of cancer cells to various chemotherapeutic agents is known as multidrug resistance (MDR), and remains a critical factor in the success of cancer treatment [1]. A key mechanism for MDR is enhanced cellular efflux of chemotherapeutic agents due to overexpression of ATP-Binding Cassette (ABC) transporters, for example ABCB1/P-glycoprotein (MDR1), the ABCC/multidrug resistance protein (MRP) family, and ABCG2/breast cancer resistance protein (BCRP) [1]–[3]. Recent studies demonstrated that ABCG2/BCRP was highly abundant in various types of solid and hematological tumors [4]. In addition, a strong correlation between ABCG2/BCRP expression and the rate of response to chemotherapy or survival was found in tumor samples from 72 non-small cell lung cancer patients [5]. Therefore, ABCG2/BCRP as well as MDR1/ABCB1 plays a significant role in drug resistance, and inhibitors for ABCG2/BCRP may enhance the outcome of cancer chemotherapy.

α-Adrenoceptor antagonists are used widely to treat hypertension, dysuria with prostatic hyperplasia, and migraine headaches [6], [7]. In addition, α-adrenoceptor antagonists used for benign prostatic hyperplasia have shown growth inhibitory effects on human prostate cancer cells [6]–[9]. Moreover, one antagonist, prazosin, was suggested to be a substrate for ABCG2/BCRP [10], [11]. However, little information is available about the effects of other α-adrenoceptor antagonists on ABCG2/BCRP.

tlb

**Figure 1:**
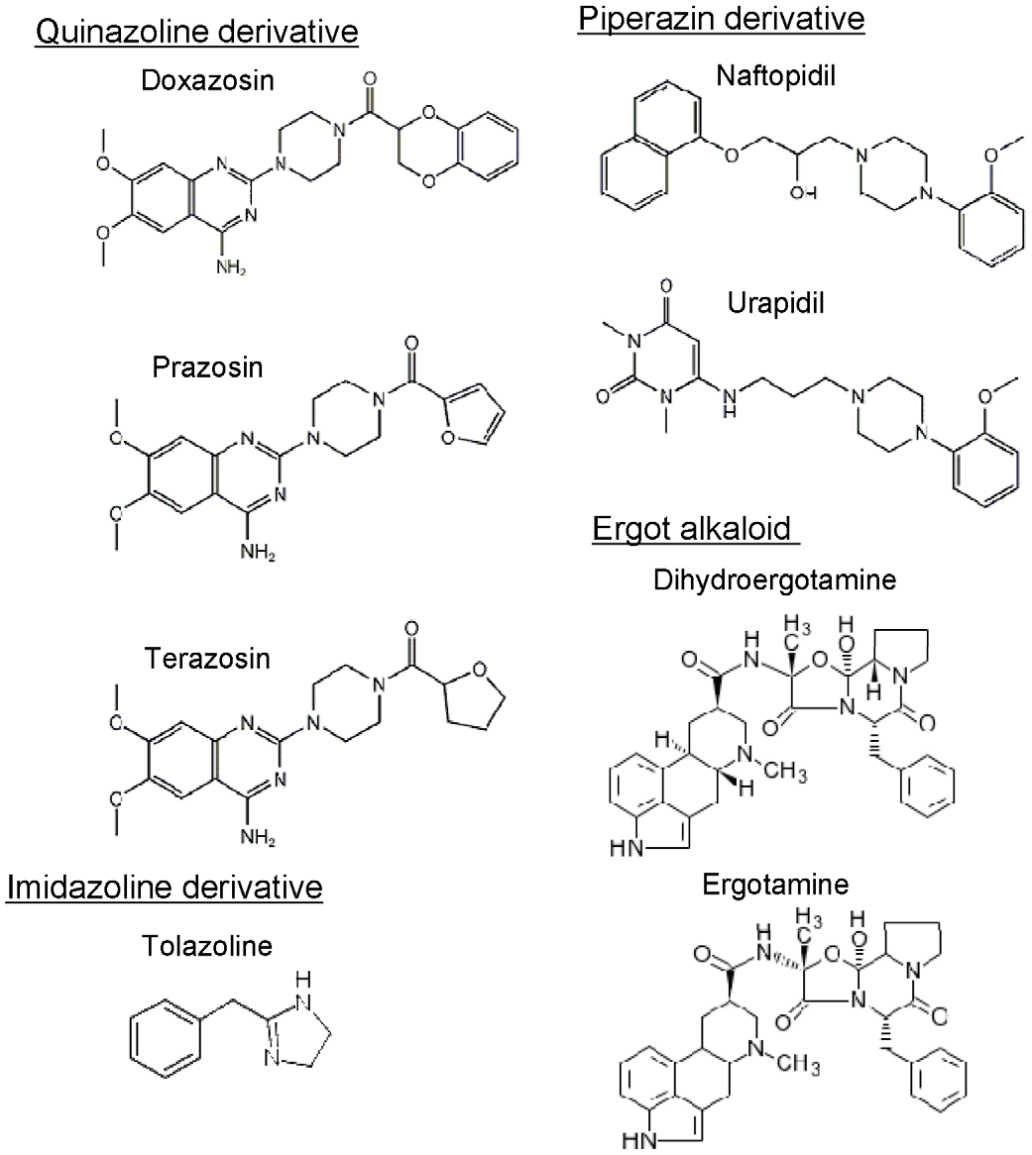
Chemical structure of α-adrenoceptor antagonists used.

## Results

### Effects of α-adrenoceptor antagonists on sensitivity to mitoxantrone

Table 1 shows the sensitivity to mitoxantrone, a substrate for ABCG2/BCRP, of HeLa and HeLa/SN100 cells in the presence of α-adrenoceptor antagonists. The IC_50_ values for mitoxantrone in HeLa cells decreased. Those in HeLa/SN100 cells exhibited a dose-dependent decrease, except for terazosin. For the cytotoxicity itself, the maximum concentration of ergot alkaloids used was 100 nM.

**Table 1:** IC_50_ values for mitoxantrone in HeLa and HeLa/SN100 cells in the presence of α-adrenoceptor antagonists.

		HeLa cells		HeLa/SN100 cells	
		IC_50_ (nM)	R.S.	IC_50_ (nM)	R.S.
Control		10.4±1.95	–	284±26.6	–
Doxazosin	0.1 µM	12.1±0.82	0.99	124±10.7*	2.40
	1 µM	6.51±0.64	1.84	64.0±7.85**	4.64
	10 µM	0.74±0.04**	16.1	7.57±1.33**	39.3
Prazosin	0.1 µM	20.1±1.95*	0.60	346±66.2	0.86
	1 µM	11.9±0.98	1.00	297±46.7	1.00
	10 µM	3.32±0.91	3.61	40.1±5.79**	7.39
Terazosin	0.1 µM	14.8±1.46	0.81	331±28.8	0.90
	1 µM	18.8±0.50*	0.64	384±61.5	0.77
	10 µM	4.66±1.44	2.57	339±47.4	0.88
Tolazoline	0.1 µM	6.77±0.58	1.77	244±40.9	1.22
	1 µM	1.59±0.55*	7.56	193±58.6	1.54
	10 µM	2.45±0.45*	4.90	45.7±6.46**	6.51
Naftopidil	0.1 µM	9.42±3.17	1.27	152±7.13	1.95
	1 µM	3.65±0.81	3.29	136±14.0*	2.19
	10 µM	2.07±0.28*	5.79	47.8±7.32**	6.22
Urapidil	0.1 µM	2.99±1.12*	4.00	127±7.82*	2.34
	1 µM	4.07±0.92	2.95	138±7.83	2.15
	10 µM	2.19±0.34*	5.48	52.1±0.85**	5.70
Dihydroergotamine	1 nM	6.71±1.26	1.79	271±46.6	1.10
	10 nM	6.07±2.11	1.98	186±14.8	1.60
	100 nM	5.23±1.26	2.29	79.8±15.4**	3.73
Ergotamine	1 nM	10.9±2.63	1.10	213±47.4	1.39
	10 nM	3.03±1.40	3.96	261±37.4	1.14
	100 nM	1.03±0.54*	11.6	52.1±8.83**	5.70

Each value shows the mean ± S.E. (n = 4).

Relative sensitivity: the ratio of IC_50_ values for mitoxantrone in the control divided by that in the groups treated with α-adrenoceptor antagonists.

*and**: significantly different from the respective control at *p*<0.05 and *p*<0.01, respectively.

### Effects of α-adrenoceptor antagonists on ABCG2/BCRP mRNA expression

**Figure 2:**
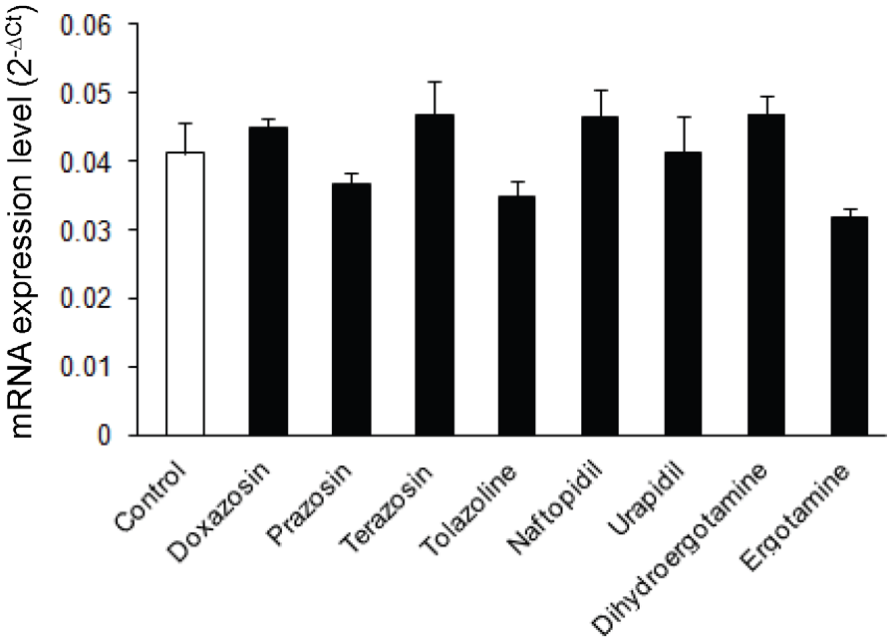
mRNA levels of ABCG2/BCRP. HeLa/SN100 cells were incubated with medium containing α-adrenoceptor antagonists (ergot alkaloids: 100 nM; other α-adrenoceptor antagonists: 10 µM) for 24 h. Total RNA was extracted from the cells, and ABCG2/BCRP mRNA expression normalized to β-actin mRNA expression was evaluated by quantitative real-time PCR. Each bar represents the mean ± S.E. (n = 3).

The level of ABCG2/BCRP mRNA was examined in HeLa/SN100 cells cultured with or without α-adrenoceptor antagonists (Figure 2). The level was not influenced by any of the antagonists.

### Effects of α-adrenoceptor antagonists on accumulation and efflux of Hoechst33342

To examine whether sensitivity to mitoxantrone depends on the activity of ABCG2/BCRP, the accumulation and efflux of Hoechst33342, a substrate for ABCG2/BCRP, in HeLa and HeLa/SN100 cells were evaluated in the absence or presence of α-adrenoceptor antagonists.

**Figure 3 pone-0030697-g003:**
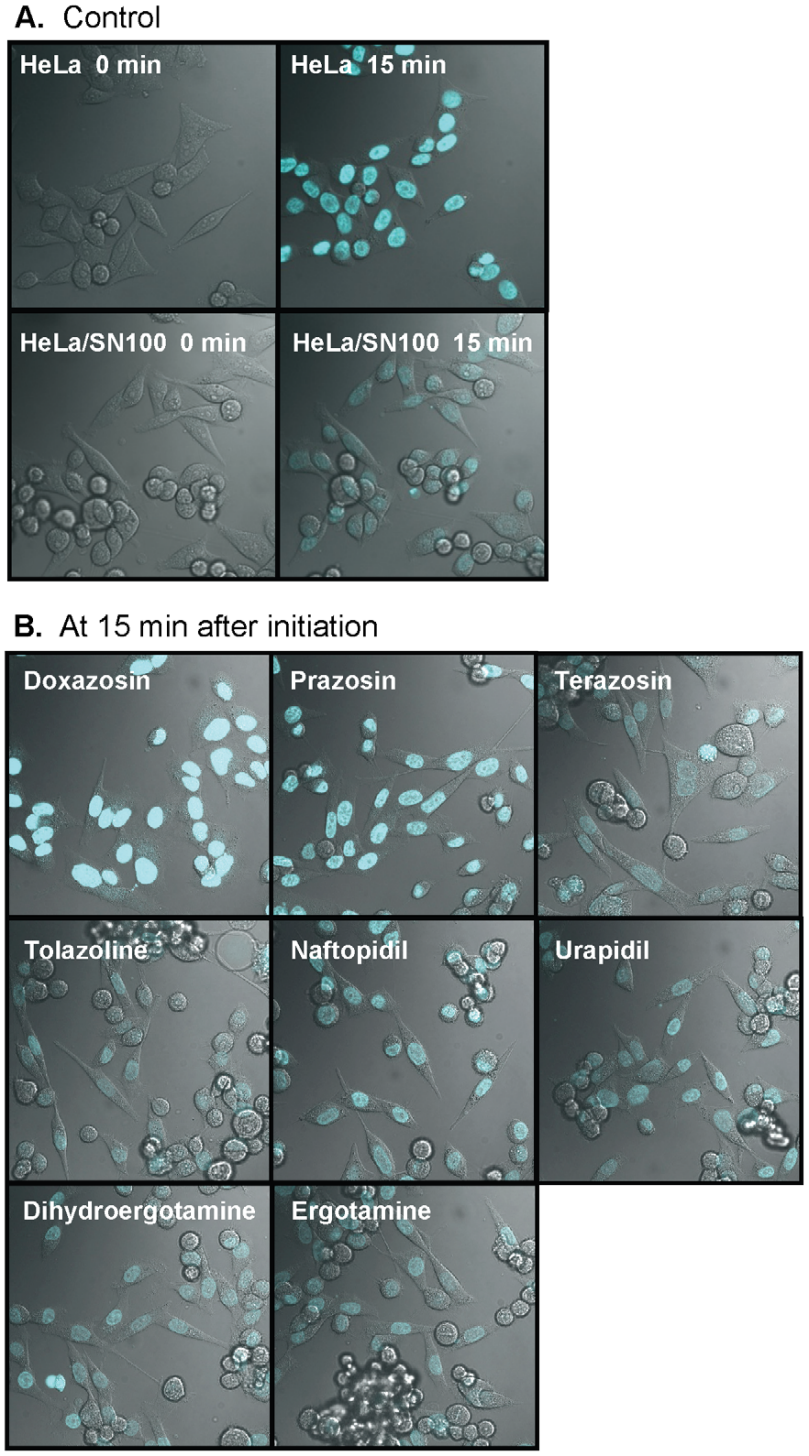
Cellular distribution of Hoehst33342. HeLa and HeLa/SN100 cells were precultured for 48 h in a humidified atmosphere containing 5% CO_2_ at 37°C. (A) HeLa and HeLa/SN100 cells were incubated with phenol red-free HBSS containing 3 µM of Hoechst33342, and images were acquired at 0 and 15 min. (B) The effects of α-adrenoceptor antagonists at 10 µM (except for ergot alkaloids at 100 nM) for 15 min were demonstrated in HeLa/SN100 cells.

Fluorescence micrographs revealed less accumulation in HeLa/SN100 cells than in HeLa cells (Figure 3). The decrease was reversed by doxazosin and prazosin, but the other α-adrenoceptor antagonists had no remarkable effect.

**Figure 4 pone-0030697-g004:**
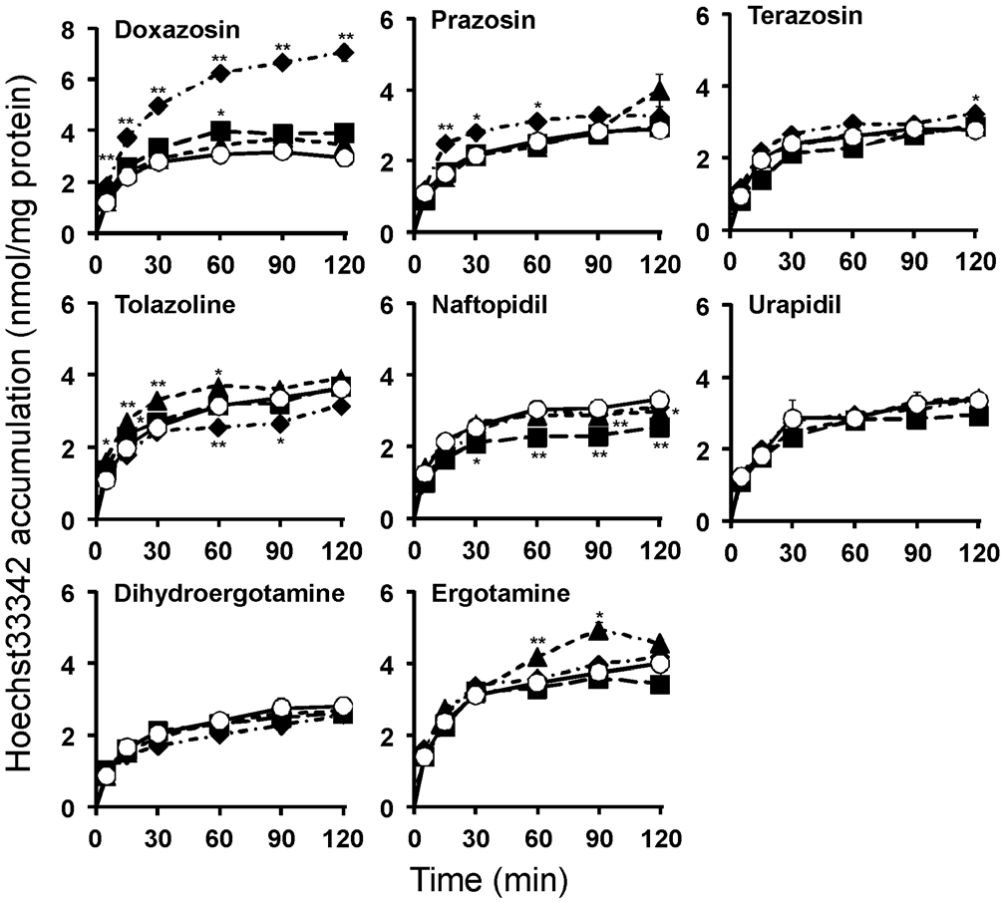
Time course of accumulation of Hoechst33342. Cells were incubated with 3 µM Hoechst33342 in the absence (○) or presence of α-adrenoceptor antagonists (ergot alkaloids: ▴, 1 nM; ▪, 10 nM; ♦, 100 nM, other α-adrenoceptor antagonists: ▴, 0.1 µM; ▪, 1 µM; ♦, 10 µM) for the desired times at 37°C. Each point represents the mean ± S.E. (n = 3), and error bars are included in the symbols. * and ** p<0.05 and 0.01 significantly different from the control at the corresponding time points, respectively.

**Figure 5:**
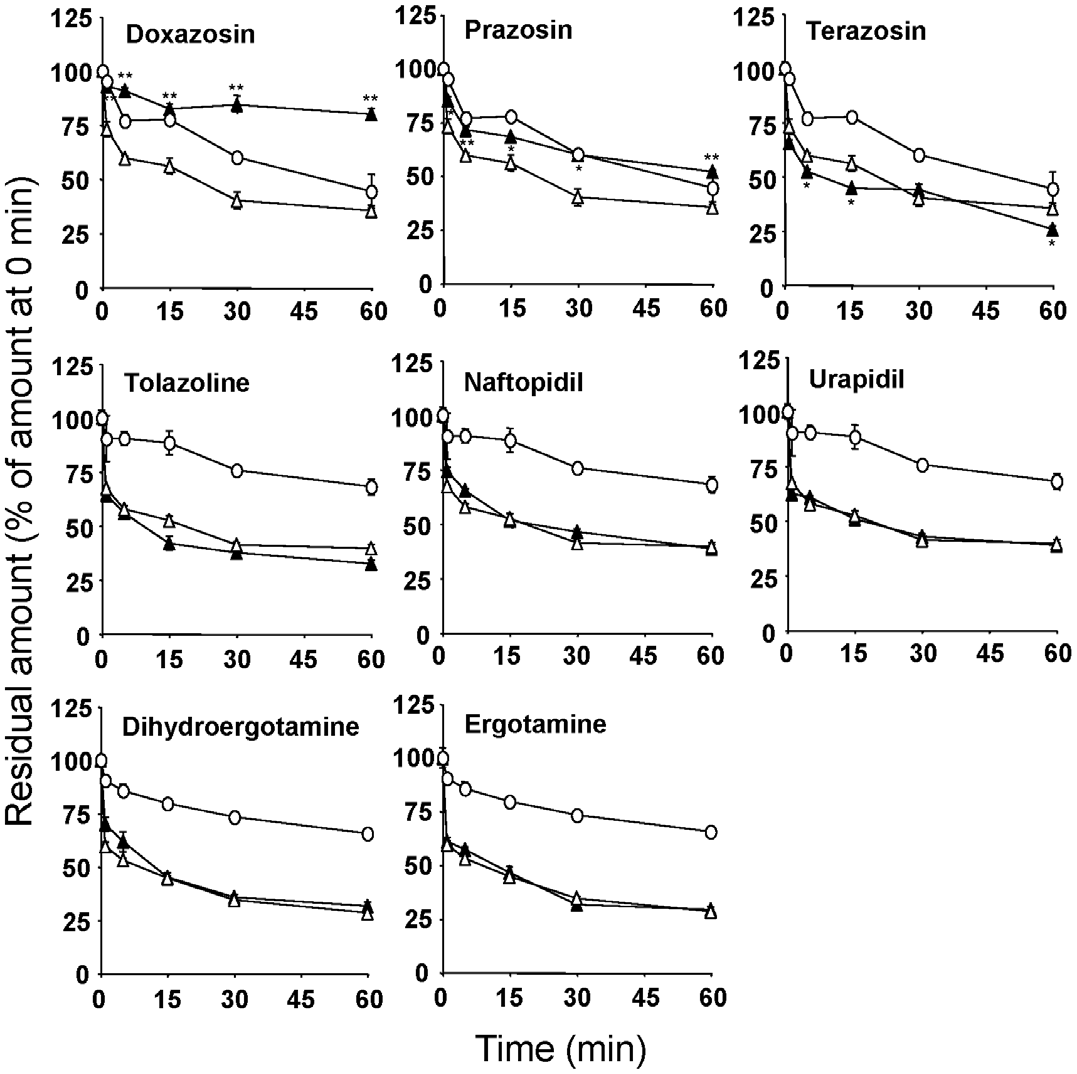
Time course of the efflux of Hoechst33342. Cells were incubated with 3 µM Hoechst33342 in the presence of 10 µM of α-adrenoceptor antagonists (100 nM for ergot alkaloids) for 60 min at 37°C, washed twice with ice-cold HBSS and incubated with warmed HBSS in the presence of 10 µM of α-adrenoceptor antagonists (100 nM for ergot alkaloids) for the desired times. ○, HeLa; •, HeLa/SN100; Δ, HeLa/SN100+10 µM α-adrenoceptor antagonists (100 nM for ergot alkaloids). Each point represents the mean ± S.E. (n = 3), and error bars are included in the symbols. * and ** p<0.05 and 0.01 significantly different from the HeLa/SN100 cells in the absence of α-adrenoceptor antagonists at the corresponding time points, respectively.

The quantitative analysis of the transport of Hoechst33342 indicated that the accumulation of Hoechst33342 in HeLa/SN100 cells was increased significantly by 10 µM of doxazosin or prazosin (Figure 4). However, the other α-adrenoceptor antagonists had no effect at any concentration. In addition, the efflux of Hoechst33342 from HeLa/SN100 cells reverted to the level for HeLa cells in the presence of 10 µM doxazosin or prazosin, but was not affected by the other antagonists (Figure 5).

### Effects of α-adrenoceptor antagonists on the cell cycle of HeLa/SN100 cells treated with mitoxantrone

The cell cycle of HeLa/SN100 cells was analyzed to explore the mechanism by which α-adrenoceptor antagonists reverse resistance to mitoxantrone. Exposure to mitoxantrone for 8 h resulted in 46.2% of cells in G_0_/G_1_ phase, 37.6% in S phase, and 16.2% in G_2_/M phase (Table 2). Concomitant administration of doxazosin, terazosin, and prazosin tended to increase the number of cells in S phase, comparable to the findings in HeLa cells treated with mitoxantrone (data not shown). In the presence of tolazoline, naftopidil, and urapidil, the distribution of G_2_/M phase cells increased. Dihydroergotamine and ergotamine did not affect the cell cycle profile in either cell type.

**Table 2:** Cell cycle profile of HeLa/SN100 cells treated with mitoxantrone in the presence of α-adrenoceptor antagonists.

	G_0_/G_1_ phase (%)	S phase (%)	G_2_/M phase (%)
Control	46.2	37.6	16.2
Doxazosin	35.0	43.7	21.3
Prazosin	30.1	48.9	21.0
Terazosin	40.3	41.4	18.3
Tolazoline	39.7	35.1	25.2
Naftopidil	35.0	40.3	24.7
Urapidil	36.5	39.9	23.6
Dihydroergotamine	43.7	37.8	18.5
Ergotamine	42.6	39.8	17.6

HeLa/SN100 cells were incubated with mitoxantrone for 8 hours in the absence (Control) or presence of α-adrenoceptor antagonists before being stained with propidium iodide. DNA content was analyzed by flow-cytometry and the percentage of cells in each phase of the cell cycle was obtained by using the Modfit program.

## Discussion

α-adrenoceptor antagonists have been reported to inhibit growth and induce apoptosis in malignant prostatic cells [13]–[17]. However, little is known about how they affect the cytotoxicity of chemotherapeutic agents. In addition, little information is available about the effects of α-adrenoceptor antagonists on ABCG2/BCRP, although prazosin was reported to be a substrate for ABCG2/BCRP.

In the present study, the sensitivity to mitoxantrone in HeLa/SN100 cells was reversed by α-adrenoceptor antagonists in a concentration-dependent manner (Table 1). In addition, such effects were found in HeLa cells with a spontaneous expression of ABCG2/BCRP [12], implying that ABCG2/BCRP-mediated transport also take place in HeLa cells. This was supported by the findings that the accumulation of Hoechst33342 in HeLa cells increased by doxazosin and prazosin (data not shown). However, levels of ABCG2/BCRP mRNA were not influenced (Figure 2), suggesting that α-adrenoceptor antagonists affect the function but not expression of ABCG2/BCRP.

Therefore, the effects of quinazoline-based α-adrenoceptor antagonists (quinazoline derivatives), i.e, doxazosin, prazosin, and terazosin, on the function of ABCG2/BCRP were examined. The transport activity of Hoechst33342 was decreased by doxazosin or prazosin, but not terazosin (Figures 3–5). Doxazosin and prazosin also increased the proportion of S phase cells among HeLa/SN100 cells (Table 2), suggesting an acceleration of the actions of mitoxantrone, i.e., S phase arrest. However, terazosin did not affect the cell cycle. These findings suggested that both doxazosin and prazosin reversed the resistance to mitoxantrone *via* the inhibition of ABCG2/BCRP-mediated transport, resulting in an acceleration of the cell cycle's arrest by mitoxantrone. In addition, terazosin little affected the function of ABCG2/BCRP, and this was supported by the absence of an effect on the cell cycle. Doxazosin, prazosin, and terazosin have the same chemical structure, carbonylpiperazino-dimethoxyquinazoline, but different side chains, i.e., benzodioxane, furan, and oxofuran, respectively. However, the three agents have similar characteristics. The reason why only terazosin did not affect ABCG2/BCRP remains unclear, and requires further study.

The other α-adrenoceptor antagonists except for the quinazoline derivatives did not affect ABCG2/BCRP-mediated transport or expression (Figures 2–5), but most of them showed the reversing effects (Table 1), suggesting that they enhance sensitivity to mitoxantrone *via* another pathway. However, these pathways remain unclear, but the followings may be considered.

Tolazoline, naftopidil, and urapidil increased the proportion of cells in the G_2_/M phase, whereas the ergot alkaloid had no effect (Table 2). These findings suggested that tolazoline, naftopidil, and urapidil sensitized cells to mitoxantrone *via* a pathway independent of transport inhibition, and their actions on the cell cycle may be involved in the enhancement of sensitivity to mitoxantrone. Naftopidil was recently suggested to inhibit the growth of human prostate cancer cells by inducing apoptosis through G_1_ arrest [18], [19]. The present findings may conflict with these previous reports [18], [19], but could be associated with novel mechanisms of cell cycle arrest by naftopidil. In the case of the ergot alkaloid, the activation of caspase-3 may contribute to the enhancement of sensitivity to mitoxantrone, since the ergot alkaloid was reported to activate caspase-3 [20]. The pattern of cell death, i.e. necrosis or apoptosis, after treatment with mitoxantrone was also reported to differ depending on the type of cell [21]. The present findings may represent the combined effects of mitoxantrone and the ergot alkaloid activating caspase-3, but it is necessary to examine the pattern of cell death after treatment with mitoxantrone in HeLa/SN100 cells.

Stimulation of the α_1_-adrenoceptor was also reported to induce cell proliferation and increase DNA synthesis in various types of cells [22], [23]. Sensitivity to mitoxantrone was enhanced by α-adrenoceptor antagonists in not only HeLa/SN100 cells but also HeLa cells. This may be achieved in part by blocking the stimulation of the α_1_-adrenoceptor.

The overlap of substrates and inhibitors between ABCB1/P-glycoprotein and cytochrome P450 3A4 is well established [24]. On the other hand, doxazosin was reported to be a more potent inhibitor for ABCB1/P-glycoprotein than prazosin [25]. This trend was also observed in the present study using ABCG2/BCRP-overexpressing cells, suggesting an overlap of substrates or inhibitors between ABCG2/BCRP and ABCB1/P-glycoprotein. The present findings supported the previous report [26].

The present findings suggested that doxazosin and prazosin reversed the resistance mainly *via* inhibition of ABCG2/BCRP-mediated transport, but the others, at least partly, affected sensitivity to mitoxantrone by acting on the cell cycle.

## Materials and Methods

### Chemicals

7-ethyl-10-hydroxycamptothecin (SN-38), an active metabolite of irinotecan hydrochloride, was a gift from Yakult Honsha Co., Ltd. (Tokyo, Japan). Doxazosin mesylate was provided by Pfizer Japan Inc. (Tokyo, Japan). Tolazoline hydrochloride was purchased from Avocado Research Chemical, Co. (Lancashire, England). Ergotamine tartrate and dihydroergotamine mesylate were purchased from Tokyo Chemical Industry Co., Ltd. (Tokyo, Japan). Prazosin and bis-benzimide H33342 trihydrochloride (Hoechst33342) were obtained from Wako Pure Chemical Industries, Ltd. (Osaka, Japan), and terazosin hydrochrolide, urapidil hydrochloride, naftopidil dihydrochloride, and mitoxantrone dihydrochloride, from Sigma-Aldrich Chemical, Co. (St Louis, MO). 2-(4-Iodophenyl)-5-(2, 4-disulfophenyl)-2H-tetrazolium, monosodium salt (WST-1) and 1-methoxy-5-methylphenazinium methylsulfate were purchased from Dojindo Laboratories (Kumamoto, Japan).

### Cells and cell culture

HeLa cells [12] were maintained in Dulbecco's modified Eagle's medium (DMEM; Invitrogen, Corp., Carlsbad, CA) supplemented with 10% heat-inactivated fetal bovine serum (lot. no. 99H2314 or 40K2368, Sigma-Aldrich) and 100 mg/L of kanamycin sulfate (Invitrogen). Cells were seeded into culture flasks, grown in a humidified atmosphere of 5%CO_2_–95% air at 37°C, and subcultured with 0.05% trypsin-0.02% EDTA (Invitrogen). HeLa/SN100 cells were established by continuous exposure to SN-38 in our laboratory [12]. They were maintained in a similar manner to HeLa cells, except that the DMEM contained 100 nM SN-38. The number of passages for HeLa and HeLa/SN100 was 398–408 and 15–24, respectively.

### WST-1 colorimetric assay

Effects of α-adrenoceptor antagonists on sensitivity to mitoxantrone in HeLa and HeLa/SN100 cells were evaluated with the WST-1 assay [27]. Cells were seeded on 96-well plates and pre-cultured for 24 h. The medium was exchanged for one containing mitoxantrone at various concentrations with or without α-adrenoceptor antagonists (ergotamine products: 1 nM, 10 nM or 100 nM; others: 0.1 µM, 1 µM or 10 µM) (Figure 1), and then cells were incubated for 72 h at 37°C. The culture medium was replaced with a medium containing a WST-1 reagent, and 3 h later, the absorbance in the well was determined at 450 nm with a reference wavelength of 630 nm by using a microplate reader (SpectraFluor™, Tecan, Switzerland). The 50% growth inhibitory concentration (IC_50_) of the α-adrenoceptor antagonists or mitoxantrone was estimated according to the sigmoid inhibitory effect model, E = E_max_×[1−C^γ^/(C^γ^+IC_50_^γ^)], using the nonlinear least-squares fitting method (Solver, Microsoft® Excel). E and E_max_ represent the surviving fraction (% of control) and its maximum, respectively. C and γ represent the concentration in the medium and the sigmoidicity factor, respectively. Relative sensitivity was defined by dividing the IC_50_ value for mitoxantrone in the control by that in the groups treated with calcium antagonists.

### Expression of ABCG2/BCRP mRNA by a real time RT-PCR assay

HeLa/SN100 cells were seeded into 60-mm dishes and pre-cultured for 24 h. The medium was exchanged for one containing α-adrenoceptor antagonists (ergotamine products: 100 nM; others: 10 µM) and cells were incubated for 24 h. Then, total RNA extracted from the cells with a GenElute™ Mammalian Total RNA Miniprep kit (Sigma-Aldrich). Total RNA (0.5 µg) was reverse transcribed with a PrimeScript™ RT reagent kit (TakaraBio, Shiga, Japan). Thermal cycling conditions were 37°C for 15 min and 85°C for 5 s as heat shock. The real-time quantitative PCR assay was performed using the Applied Biosystems 7500 Sequence detecting system (Applied Biosystems, Foster City, CA), and SYBR® Premix *Ex Taq*™ II (TakaraBio). cDNA (20 ng per sample) was used to perform duplicate PCR analyses. The primers for amplification were as follows: ABCG2/BCRP-Forward; TGA CGG TGA GAG AAA ACT TAC, ABCG2/BCRP-Reverse; TGC CAC TTT ATC CAG ACC T, β-actin-Forward; TCA TGA AGT GTG ACG TGG ACA TC, β-actin-Reverse; TGC ATC CTG TCG GCA ATG. The threshold cycle (Ct) was used for determining the relative expression level of each gene, by normalizing to the Ct of β-actin. The ΔΔCt was used to calculate the relative change. The equation for analyzing ABCG2/BCRP expression was as follows: ABCG2/BCRP level = 2^−ΔCt^, where ΔCt = Ct (ABCG2/BCRP)−Ct (β-actin). A dissociation curve analysis was also performed at the end of the amplification process, because SYBR® Green binding was not sequence specific.

### Fluorescent imaging

HeLa and HeLa/SN100 cells were seeded on a 35-mm glass-bottom culture dish (Matsunami Glass Ind., Ltd. Osaka Japan), and pre-cultured for 48 h in a humidified atmosphere of 5% CO_2_–95% air at 37°C. Cells were washed twice with warmed Hanks' balanced salt solution containing 25 mM HEPES (HBSS), and incubated with 3 µM of Hoechst33342 in the absence or presence of α-adrenoceptor antagonists (ergotamine products: 100 nM; others: 10 µM) for 15 min at 37°C. Fluorescence micrographs were acquired at 0 and 15 min using a confocal laser scanning microscope (LSM510META Ver4.2, Carl Zeiss Co., Ltd, Oberkochen Germany) with a blue diode for excitation (405 nm).

### Transport experiments with Hoechst33342

In the accumulation experiments, HeLa and HeLa/SN100 cells were seeded on 24-well plates, and pre-cultured for 48 h in a humidified atmosphere of 5% CO_2_–95% air at 37°C. The cells were washed three times with warmed HBSS, and the accumulation experiments were started with the addition of fresh HBSS containing 3 µM Hoechst33342, a substrate for ABCG2/BCRP, with or without α-adrenoceptor antagonists (ergotamine products: 1 nM, 10 nM, or 100 nM; others: 0.1 µM, 1 µM, or 10 µM).

In the efflux experiments, HeLa and HeLa/SN100 cells were pre-cultured as described for the accumulation experiments. Cells were washed three times with warmed HBSS and pre-loaded with the incubation medium in fresh HBSS containing 3 µM Hoechst33342 with or without α-adrenoceptor antagonists (ergotamine products: 100 nM; others: 10 µM) for 60 min. After being loaded, HBSS was immediately removed and cells were washed rapidly twice with ice-cold HBSS. Efflux experiments were started with the addition of fresh warmed HBSS with or without α-adrenoceptor antagonists (ergotamine products: 100 nM; others: 10 µM), and further incubated at 37°C. In both experiments, the reaction was stopped at the desired times by aspirating the HBSS from the well, followed by washing three times with ice-cold phosphate-buffered saline (PBS). Then, cells were lysed with PBS containing 0.1% Tween20®. Aliquots (100 µL) of the cell lysate were diluted twice and transferred to 96-well black plates, and the fluorescence intensity of Hoechst33342 was measured with an excitation wavelength of 340 nm and emission wavelength of 465 nm using SpectraFluor™ (Tecan). Protein content was determined by the Lowry method [28] using bovine serum albumin (Sigma-Aldrich) as the standard.

### Flow cytometry

HeLa and HeLa/SN100 cells were seeded on 60-mm dishes and pre-cultured for 24 h. The medium was exchanged for one containing 50 nM mitoxantrone in the absence or presence of α-adrenoceptor antagonists (ergotamine products: 100 nM; others: 10 µM), and cells were incubated for 8 h. Cells were trypsinized and washed with ice-cold PBS, and fixed in 70% ethanol containing PBS. The harvested cells were treated with propidium iodide (PI) staining buffer containing RNase (Becton-Dickinson, San Jose, CA, USA) for 15 min, and analyzed by a flow cytometer (FACSCalibur HG™, Becton-Dickinson). PI was detected with 488 nm excitation, and a 564–606 nm band pass filter.

### Statistical analysis

Comparisons were performed with a non-repeated one-way analysis of variance followed by the Dunnett test for multiple comparisons. A *p* value of less than 0.05 (two-tailed) was considered significant.
